# Huge Gap Between Clinical Efficacy and Community Effectiveness in the Treatment of Chronic Hepatitis C: A Nationwide Survey in Taiwan

**DOI:** 10.1097/MD.0000000000000690

**Published:** 2015-04-03

**Authors:** Ming-Lung Yu, Ming-Lun Yeh, Pei-Chien Tsai, Ching-I. Huang, Jee-Fu Huang, Chung-Feng Huang, Meng-Hsuan Hsieh, Po-Cheng Liang, Yi-Hung Lin, Ming-Yen Hsieh, Wen-Yi Lin, Nai-Jen Hou, Zu-Yau Lin, Shinn-Cherng Chen, Chia-Yen Dai, Wan-Long Chuang, Wen-Yu Chang

**Affiliations:** From the Hepatobiliary Division, Department of Internal Medicine and Hepatitis Center, Kaohsiung Medical University Hospital (Ming-Lung Yu, Ming-Lun Yeh, P-CT, J-FH, C-FH, P-CL, Y-HL, M-YH, Z-YL, S-CC, C-YD, W-LC, W-YC); Faculty of Internal Medicine, College of Medicine, and Graduate Institute of Clinical Medicine, and Center for Infectious Disease and Cancer Research, Kaohsiung Medical University (Ming-Lung Yu, J-FH, C-FH, M-HH, Z-YL, S-CC, C-YD, W-LC); Institute of Biomedical Sciences, National Sun Yat-Sen University (Ming-Lung Yu); Department of Internal Medicine, Kaohsiung Municipal Hsiao-Kang Hospital, Kaohsiung Medical University (C-IH, N-JH); Department of Occupational Medicine, Kaohsiung Municipal Ta-Tung Hospital (C-FH); Department of Preventive Medicine, Kaohsiung Medical University Hospital (M-HH); Department of Occupational Medicine, Kaohsiung Municipal Hsiao-Kang Hospital, Kaohsiung Medical University (W-YL); and Taiwan Liver Research Foundation, Kaohsiung, Taiwan (W-YC).

## Abstract

Peginterferon/ribavirin provides a substantially high treatment efficacy for chronic hepatitis C virus (HCV) infections in Asians. Whether the clinical efficacy can be translated to community effectiveness remains unclear.

The disease awareness, treatment accessibility, recommendations, acceptance, and barriers to anti-HCV treatment were explored to clarify the issue with a 3-step nationwide investigation in Taiwan. A crude HCV-infected population was estimated using databases from 3 large-scale surveillance studies and age-/geographic-specific population database. HCV awareness and accessibility were investigated at the patient level in 58,129 residents. The recommendations/acceptances and barriers to treatment at the provider level were evaluated using a prospective, nationwide approach to 89 gastroenterologists/hepatologists.

The estimated 10-year interval age-adjusted anti-HCV-seropositive population is 745,109 (3.28%), with an anticipated HCV-viremic population of 554,361. Of anti-HCV-seropositive subjects, 36.2% had disease awareness. Among those with awareness, 39.6% had accessibility. The recommendation/acceptance rate of antiviral therapy was 70.6%. The treatment rate was 10.1% and 13.7% for the anti-HCV-seropositive and HCV-viremic population, respectively. With an anticipated treatment success rate of 80% in Taiwan, 8.1% of the anti-HCV-seropositive and 10.9% of the HCV-viremic population achieved successful treatment. The major treatment barriers were fear of adverse effects (37%), major disorders (17.6%), ineligibility for insurance reimbursement (17.6%), and lack of therapy awareness (11.3%).

Despite the high rates of treatment response and nationwide coverage of insurance reimbursement, there remains a large gap between clinical efficacy and community effectiveness in anti-HCV treatment in Taiwan. Increasing disease awareness/treatment accessibility and introducing new therapeutic strategies with high tolerability are warranted.

## Abstract 2

Supplemental Digital Content is available in the text

## BACKGROUD

Hepatitis C virus (HCV) infection is one the leading causes of chronic hepatitis, liver cirrhosis, and hepatocellular carcinoma (HCC) worldwide.^1,2^ HCV infection is endemic in Taiwan, with the prevalence rates of antibodies to HCV (anti-HCV) ranging from 4.4% to 8.6%; however, there are scattered hyperendemic areas in Southern Taiwan that have a very high anti-HCV prevalence of 22.4%.^3,4^ Peginterferon (PegIFN) along with ribavirin (RBV) combination therapy has been the standard of care for patients with chronic hepatitis C for more than a decade,^1^ and it remains the mainstay of current anti-HCV therapy in the majority of Asian countries where interferon (IFN)-free direct acting antivirals (DAAs) regimens are unavailable or unaffordable. Chronic HCV infection (CHC) has become a curable disease^5^ through the achievement of a sustained virological response (SVR), which leads to a significantly reduced risk of cirrhosis, HCC and mortality.^6–9^ The treatment efficacy is particularly remarkable in Asian patients. A 48- or 24-week regimen of PegIFN/RBV could attain an SVR rate of 70% to 75% or 85% to 90% for HCV genotype 1/4 (HCV-1/4) and HCV-2/3, respectively, in Taiwan.^10–13^ The achievement of high SVR rates encourages HCV control in Taiwan.

There might be a gap between real-world daily practice and the promising results obtained in clinical trials because of low disease awareness, difficulty in treatment accessibility, and contraindications and adverse events (AEs) from available treatment modalities. The concept of community effectiveness demonstrates the effect of biomedical research and pharmacological development on human health in the community at large.^14^ It is important to determine whether the dramatic efficacy from clinical trials could be translated to community effectiveness to assess the overall effectiveness. There remain many barriers leading to a significant proportion of HCV patients who have not received antiviral therapy.^15^ Fewer than half of HCV subjects were aware of their infections or had been identified as having anti-HCV-seropositive in the United States.^15–17^ Only two-thirds of anti-HCV-identified individuals received a complete diagnosis that included tests for HCV RNA.^18^ Fewer than one-third of HCV-viremic patients had started antiviral treatment.^17^ Barriers to treatment include economic or social pressure, fear of AEs, fear of needle injection, and reimbursement policies.^19–23^ Patients with preexisting comorbid conditions such as major systemic disorders, psychiatric illnesses, neutropenia, and thrombocytopenia might be ineligible for current IFN-based therapy.

More than half of HCV-infected individuals worldwide reside in Asia, which has an anti-HCV prevalence rate of >3.5%.^2^ However, data about HCV disease awareness, treatment accessibility, and recommendation/acceptance are scarce. Based on the high treatment efficacy of PegIFN/RBV in the Asian population^10^ and the upcoming new therapeutic approaches with potent DAA,^1,24–29^ understanding the barrier between community effectiveness and clinical efficacy would help public health decision makers bridge the gap to control HCV infection. We constructed a model for nationwide translational research at the patient, provider, and government levels on the community effectiveness of anti-HCV therapy to explore disease awareness, treatment accessibility, and barriers to clarify these issues in Taiwan.

## METHODS

### Estimation of HCV Prevalence and Population

We estimated a national age-specific prevalence and population of HCV infection based on the data obtained in three large-scale surveillance studies for hepatitis in Taiwan^3,4,30^ and an age-/geographic-specific population information of Taiwan from the Ministry of the Interior (http://statis.moi.gov.tw/micst).

### Estimation of Community Effectiveness

We proposed an equation modified from the conceptual framework for translating efficacy to effectiveness^14^ to estimate the nationwide community effectiveness: Community Effectiveness = (1) Correct Diagnosis × (2) Awareness × (3) Access × (4) Recommendation × (5) Acceptance × (6) Efficacy × (7) Adherence.

#### (1) Estimation of Correct Diagnosis for HCV Infection

The chance of a false-negative anti-HCV result (anti-HCV negative in HCV viremic patients) using third-generation anti-HCV antibody testing is extremely low among HCV-exposed subjects, even in immune compromised patients, such as uremic patients on maintenance hemodialysis and patients with HIV coinfection.^31,32^ We assumed that the current anti-HCV testing methods have no false negatives for subjects with HCV-exposure >6 months. According to our previous large-scale studies, the persistent HCV viremic rate among anti-HCV-positive subjects is assumed to be 74.4%.^33,34^

#### (2) Disease Awareness and (3) Disease Accessibility at Patient Level

The disease awareness of HCV infection was investigated at the patient level through serological tests and questionnaire interviews in a series of multidisciplinary community survey in Taiwan between 2012 and 2013. The disease accessibility of HCV infection was investigated at the patient level through questionnaire interviews for those with HCV disease awareness.

#### (4) Recommendation and (5) Acceptance of Anti-HCV Therapy at Provider Level

To simplify the structure, we merged the rate of treatment recommendations by physicians and the rate of acceptance of anti-HCV therapy by patients as the rate of receiving anti-HCV therapy. The treatment rate was evaluated based on the patient and provider levels to reduce potential bias. The antiviral treatment rate at the patient level was obtained by questionnaire interviews in a community survey. The anti-HCV treatment rate at the provider level was explored by a prospective, nationwide approach of sampling 89 gastroenterologists/hepatologists selected in proportion to the geographical differences in the HCV population in Taiwan (Supplementary Figure S1, http://links.lww.com/MD/A234). The treatment barriers and their characteristics in clinical practice were evaluated at the provider level. These qualified physicians were invited to participate in this clinic-based survey in December 2012 to collect information regarding their consecutive outpatients with HCV infection, including sex, age, history of anti-HCV therapy and reasons for not receiving antiviral therapy from the physicians’ point of view regarding their patients.

#### (6) Efficacy and (7) Adherence of Anti-HCV Therapy in Taiwan

We estimated the overall SVR rate of anti-HCV therapy in Taiwan according to the published data and weighted by the distribution of the HCV genotype 1 and the non-1 genotypes. The reported SVR rate was approximately 74% to 77% for HCV-1 with 48 weeks of PegIFN/RBV^5,13,35,36^ and 86% to 95% for HCV-non-1 with 24 weeks of PegIFN/RBV^5,12,37,38^ in Taiwan. The genotype distribution was 53% and 47% for HCV-1 and -non-1, respectively.^10^ Therefore, we assumed an overall efficacy of 80% for anti-HCV therapy with the current available regimens in Taiwan. To simplify the estimation, we assumed the adherence in clinical practice to be the same as the adherence observed in clinical trials.

The study was approved by the ethics committees at the Kaohsiung Medical University Hospital and was performed according to the guidelines of the International Conference on Harmonization for Good Clinical Practice. All of the residents and the interviewed gastroenterologists/hepatologists provided written informed consent before study enrollment.

### Laboratory Examinations

The anti-HCV antibody was detected using a third-generation, commercially available enzyme-linked immunosorbent assay kit (AxSYM 3.0; Abbott Laboratories, Chicago, IL). The presence of serum HCV RNA was evaluated using a standardized automated quantitative reverse-transcription polymerase chain reaction (COBAS AMPLICOR HCV Test, version 2.0; Roche, Branchburg, NJ; detection limit: 50 IU/mL) before 2011 or a real-time polymerase chain reaction assay (RealTime HCV; Abbott Molecular, Des Plaines IL; detection limit: 12 IU/mL) after 2011.^39^

### Policy of Reimbursement for Anti-HCV Therapy in Taiwan

The Bureau of National Health Insurance in Taiwan has provided reimbursement for anti-HCV therapy since 2003. The current criteria for reimbursement are seropositivity for anti-HCV and HCV RNA for >6 months with an elevated alanine aminotransferase level.

### Statistical Analysis

The estimated HCV population in a geographic-specific area was calculated as the anti-HCV-positive rate of the specific age multiplied by the total population. The rate of subjects receiving antiviral therapy was weighed by the frequency of outpatient visits for hepatitis C-related liver diseases, assuming that patients with successful antiviral therapy had visits to gastroenterology/hepatology clinics every 6 to 12 months, whereas the patients who failed prior antiviral therapy had visits to gastroenterology/hepatology clinics every 3 to 6 months, with a ratio of 1:2. The Cochran-Armitage trend test was used to identify linear relationships between the patient/clinic characteristics and the reasons for not receiving antiviral therapy. Multiple logistic analyses with adjustments for age, sex, clinic scales, and geographic areas for each reason were also performed. A 2-tailed *P* value <0.05 was considered statistically significant. Statistical analyses were performed using JMP software version 10.0 (SAS Institute Inc, Cary, NC).

## RESULTS

### Estimated National HCV Prevalence and Population

Table 1 shows the crude and age-specific prevalence of anti-HCV in each geographic-specific area in Taiwan based on 3 large-scale surveillance studies^3,4,30^ and the database of age population in 2012 from the Ministry of the Interior, Taiwan. The estimated 10-year interval age-adjusted anti-HCV prevalence was highest in Maoli County (crude: 10.5% and age-specific: 9.2%), followed by Chiayi County (crude: 10.3% and age-specific: 8.3%). Finally, the age-adjusted prevalence of anti-HCV of Taiwan was estimated to be 3.28%, with an anti-HCV-seropositive population of 745,109.

**TABLE 1:**
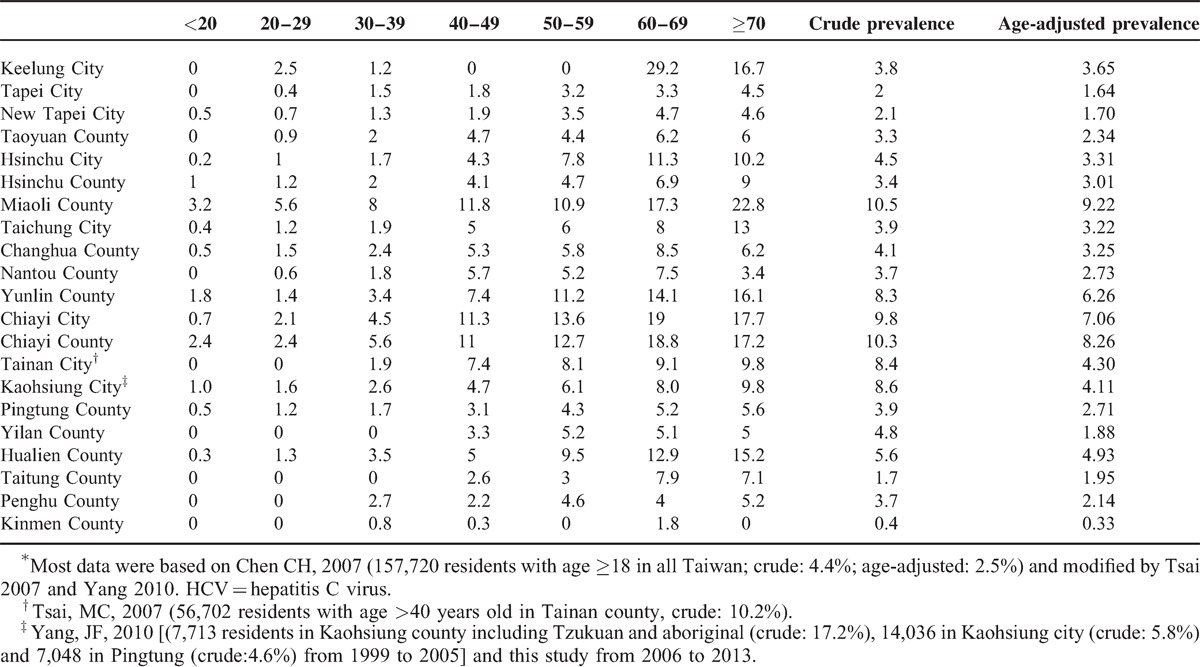
The estimated crude and age-specific prevalence (%) of HCV in Taiwan (N = 745 109; 3.28%)

Under the assumption of 100% diagnosis rate of anti-HCV^31^ and a 74.4% HCV viremic rate in the HCV-exposed subjects,^34^ the national HCV viremic population was estimated to be 554,361 in Taiwan.

### Disease Awareness

**FIGURE 1:**
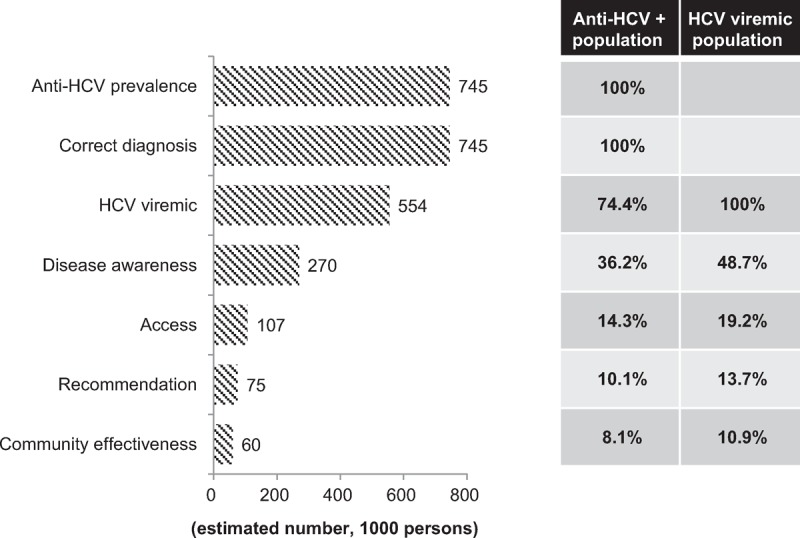
Estimated number of HCV population and community effectiveness of anti-HCV therapy in Taiwan. The numbers of anti-HCV-seropositive population, HCV-viremic population, patients with disease awareness, disease accessibility, recommendation and acceptance of antiviral therapy and successful antiviral therapy (community effectiveness) were listed on the left side. The percentages of each category among anti-HCV-seropositive population and among HCV-viremic population were listed on the right side. (A) Diagnosis of HCV infection by anti-HCV and HCV RNA were assumed at 100%. (B) Clinical efficacy with adjustment for treatment adherence was set at 80%. (C) Retreatment of HCV was not included in the current model. (D) Each percentage was calculated as the number divided by anti-HCV-seropositive population or HCV viremic population, respectively.

Of 586 anti-HCV seropositive subjects with available information, 212 (36.2%) had HCV disease awareness. Therefore, the estimated population is 269,729 for HCV disease awareness in Taiwan (Figure 1).

### Disease Accessibility

Of the 531 subjects who were aware of HCV infection and had available information regarding accessibility, 210 (39.6%) had accessibility to a doctor. Therefore, the estimated population is 106,813 for HCV disease accessibility in Taiwan (Figure 1).

### Treatment Rate of HCV

The rate of receiving anti-HCV therapy was 50.0% (105/210) in the community-based patient survey, which was comparable with 54.8% (1,668/3,043) in the clinic-based physician survey. A weighted average of 54.5% (1,773/3,253) treatment rate was further adjusted by the proposed frequency of visits (Supplementary Table S1, http://links.lww.com/MD/A234). Eventually, 70.6% of patients with HCV treatment accessibility were expected to have undergone IFN-based therapy, accounting for 75,410 patients in Taiwan (Figure 1 and Supplementary Table S1, http://links.lww.com/MD/A234).

### Successful Anti-HCV Therapy

With anticipated SVR rate of 80% for HCV patients in Taiwan, it was estimated that 60,328 HCV patients have been successfully treated (Figure 1 and Supplementary Table S1, http://links.lww.com/MD/A234).

### Gap Between Clinical Efficacy and Community Effectiveness of HCV Treatment

Among the anti-HCV-seropositive population, 36.2% (269,729/745,109) had HCV disease awareness, 14.3% (106,813/745,109) had HCV treatment accessibility (39.6% of those with HCV disease awareness), and 10.1% (75,410/745,109) had explored antiviral therapy (70.6% of those with treatment accessibility). Therefore, even with the anticipated high SVR rate of 80% for HCV patients in Taiwan, it was estimated that only 8.1% (60,328/745,109) of anti-HCV-seropositive patients have been successfully treated (Figure 1 and Supplementary Table S1, http://links.lww.com/MD/A234).

Further evaluation of community effectiveness by focusing on the HCV viremic population (n = 554,361), 74.4% of anti-HCV-seropositive population showed that the rates of disease awareness, treatment accessibility, and recommendation/acceptance of antiviral therapy were 48.7% (269,729/554,361), 19.2% (106,813/554,361), and 13.7% (75,410/554,361), respectively. We could only translate the 80% clinical efficacy into 10.9% (60,328/554,361) community effectiveness in Taiwan with the current available anti-HCV regimens (Figure 1 and Supplementary Table S1, http://links.lww.com/MD/A234).

### Treatment Barriers

**FIGURE 2:**
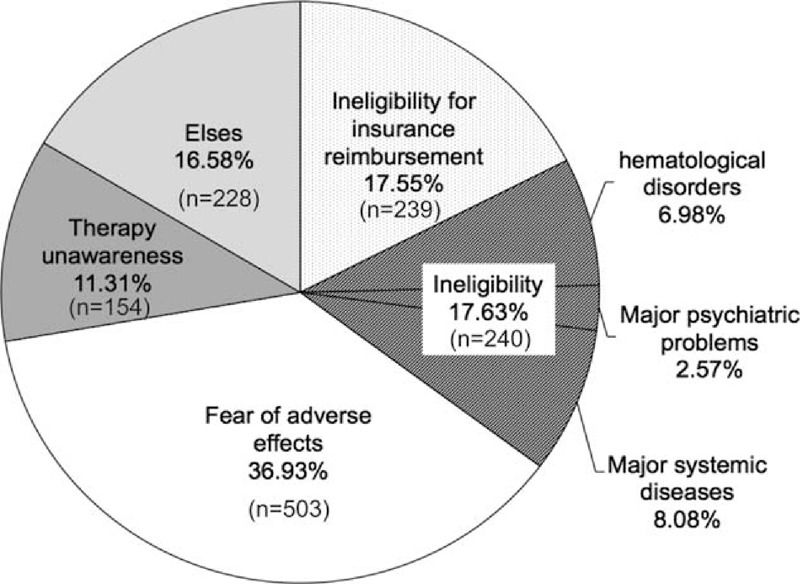
Causes for not being treated with anti-HCV therapy in clinics (n = 1,375).

Of 3,045 patients approached by questionnaire of 89 gastroenterologists/hepatologists, 1,375 were untreated (Table 2). The elderly patients (>65 years old), female patients, patients at primary clinic units, and patients residing in the central part of Taiwan had significantly higher rates of not being treated at 55.5% (503/907), 49.2% (757/1,539), 69.2% (247/357), and 51.8% (247/477), respectively, compared with their counterparts (Table 2). The main reasons for not being treated were patients’ fear of AEs (n = 503, 36.9%), major hematological disorders or systemic disorders (n = 240, 17.6%), ineligibility for insurance reimbursement (n = 239, 17.6%), and patients’ lack of therapy awareness (n = 154, 11.3%) (Figure 2).

**TABLE 2:**
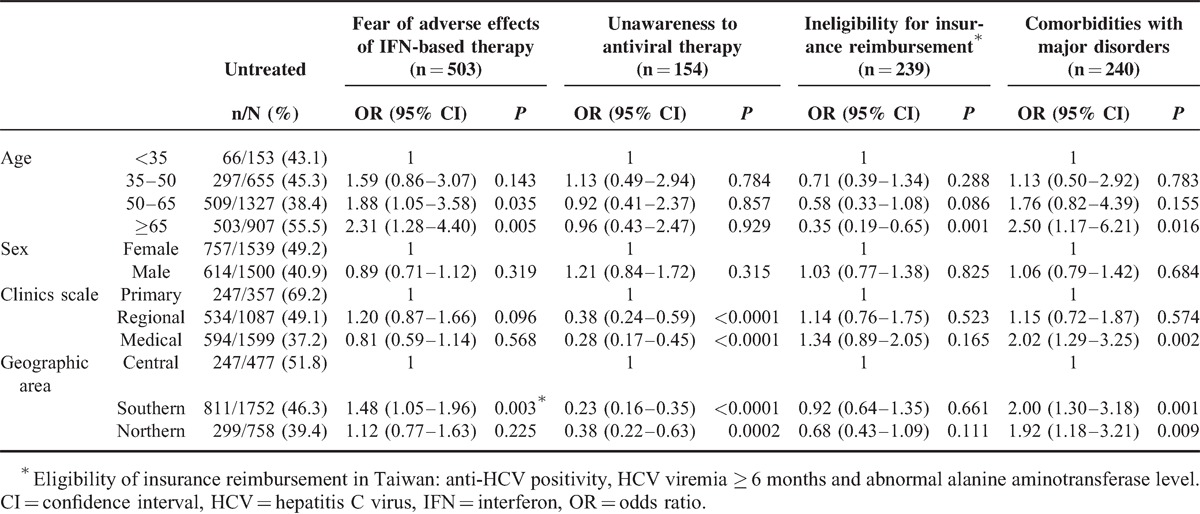
Multivariate effects of the reasons why subjects have not yet received antiviral therapy (n = 1375)

**FIGURE 3:**
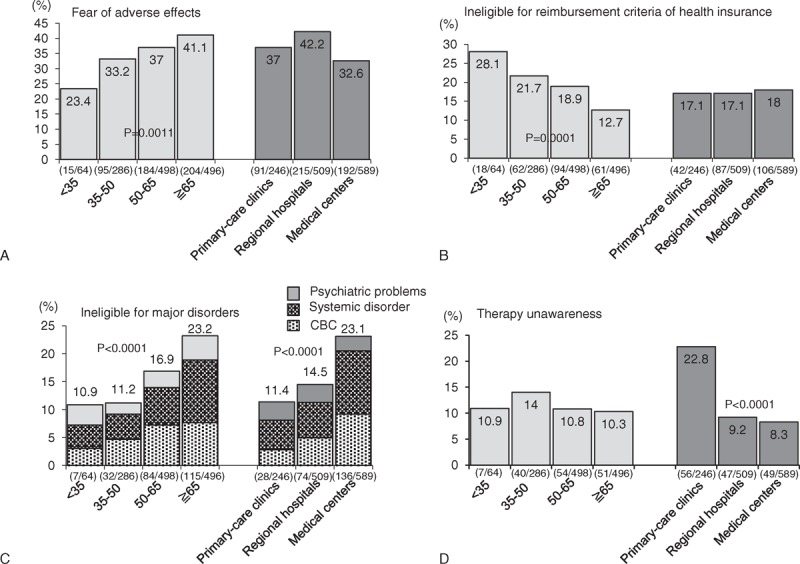
The reasons for not receiving antiviral therapy by age and clinic characteristics. (A) Fear of adverse effects. (B) Ineligible for insurance reimbursement. (C) Ineligible for major disorders. (D) Therapy unawareness. ^∗^ Adjusted *P* value: the effects of age/clinic characteristics for those reasons without receiving antiviral therapy after adjustment for risk factors. ^†^ The number (n/N) was showed in the parentheses.

The elderly patients had a 2.3-fold higher proportion of patient concern for fear of AEs (OR = 2.31 and adjusted *P* for trend test = 0.001) (Figure 3A andTable 2), a 0.35-fold lower proportion of ineligibility for insurance reimbursement criteria of HCV therapy (OR = 0.35 and adjusted *P* for trend test <0.0001) (Figure 3B and Table 2) and a 2.5-fold higher proportion of major systemic disorders than their younger counterparts (OR = 2.50 and adjusted *P* for trend test <0.0001; Figure 3C and Table 2), especially in major hematological disorders (leukopenia, anemia, or thrombocytopenia) (*P* = 0.031) and systemic disorders (such as major psychological disorders, poorly controlled diabetes, and cardiovascular diseases) (*P* < 0.0001).

More patients with ineligibility because of hematological disorders or systemic disorders were found in medical centers, followed by regional hospitals and primary care clinics (adjusted *P* for trend test = 0.002 for hematological disorders and *P* = 0.006 for systemic disorders) (Figure 3C and Table 2). We also found that patients from primary care clinics had significantly lower awareness regarding the benefits of HCV infection treatment (adjusted *P* for trend test <0.0001) (Figure 3D and Table 2).

## DISCUSSION

In the current study, we built a successful model to translate the clinical efficacy into the nationwide community effectiveness for anti-HCV therapy at the patient level, provider level, and payer level in Taiwan. Despite the high SVR rate, with an average of 80% achieved with PegIFN/RBV, and despite the very high coverage rate of insurance reimbursement in Taiwan, it is estimated that only 10.9% of the HCV-viremic population was successfully treated. Therefore, approximately 494,033 HCV-viremic patients remain at a high risk for end-stage liver disease and urgently need to be treated in Taiwan. The huge gap between the clinical efficacy and community effectiveness is largely attributed to insufficient disease awareness, poor patient accessibility to treatment, and patients who are intolerant/ineligible for PegIFN/RBV therapy. The current finding might be translated to some other Asian countries where the reimbursement policy and the frequency of favorable interleukin-28B genotype were similar to that of Taiwan.^10,40^

There are few published studies regarding the community effectiveness of HCV treatment worldwide. Supplementary Table S2, http://links.lww.com/MD/A234 summarizes the reports from the United States, the United Kingdom, and Germany, with an estimated community effectiveness of 1.1% to 8.7%.^16,17,20,41^ We observed that the community effectiveness of anti-HCV treatment was substantially higher in Taiwan than it was in Western countries. This result could be because of the high coverage rate of the National Health Insurance Program,^42^ the universal reimbursement therapy for HCV-viremic patients with abnormal alanine aminotransferase levels, and the high clinical efficacy of PegIFN/RBV.^10,12,13^ The high frequency of interleukin-28B favorable genotype (>80%)^35,38,43^ might contribute to the high SVR rate of PegIFN/RBV in Taiwan compared with in Western countries. Nevertheless, the gap between clinical efficacy and community effectiveness in HCV treatment remains large in Taiwan, indicating that efforts are needed to improve the community effectiveness.

Because there are many HCV-hyperendemic areas at the southwestern coast of Taiwan,^3,4^ several multidisciplinary integrated programs for the prevention and control of viral hepatitis have been conducted by governmental and nongovernmental organizations in recent years. These efforts have reduced the prevalence of anti-HCV seropositivity from 4.5% to 0.7% in the adult population^44,45^ and from 2.8% to 1.0% in the teenage population of the HCV-hyperendemic areas.^46,47^ Although several integrated educational programs for the prevention/treatment of viral hepatitis have been conducted by government and nonprofit organizations in Taiwan, two-thirds of anti-HCV seropositive subjects remain lack disease awareness. A low disease awareness of HCV infection of 20% to 50% was also reported in Western countries (Supplementary Table S2, http://links.lww.com/MD/A234).^16,17,20,41^ Because the majority of HCV patients are asymptomatic and the disease is curable, the situation emphasizes the critical need of governments to implement surveillance programs, especially in high-risk populations,^1,48^ and emphasizes the importance of disseminating information and increasing public and provider awareness. Although patient and provider factors receive the greatest attention, obstacles arising at the government and payer levels are also important.^49^ Successful antiviral therapy could profoundly reduce the risk of end-stage liver disease.^6–8^ However, HCV patients remain at a higher risk of HCC development even after curative HCV therapy if they had advanced fibrosis at baseline and/or after treatment.^7,50^ Therefore, the early identification of HCV viremic subjects who should receive antiviral therapy to reduce the risk and healthcare costs of liver complications is a critical public issue worldwide.

In this study, 39.6% of the subjects with HCV disease awareness had accessibility to HCV treatment in Taiwan, indicating that 14.3% of anti-HCV seropositive subjects and 19.2% of HCV-viremic subjects had seen a doctor for HCV infection. The National Health Insurance Program of Taiwan has a very high coverage rate of 99.6% of the national population^42^ and is ranked first on a list of the 10 best health care systems in the world.^51^ IFN-based therapy for CHC has been reimbursed by the Agency of National Health Insurance of Taiwan since 2003. However, the reimbursement of anti-HCV therapy is limited to HCV viremic patients with abnormal liver function by licensed gastroenterologists only. This restriction might limit treatment accessibility of patients in a country in which medical accessibility and the insurance coverage rate is extremely high.

The rate of recommendation/acceptance for HCV treatment was estimated at approximately 70% after adjusting for the frequency of visits to gastroenterologist/hepatologist clinics in this study. High treatment efficacy and insurance reimbursement for 24–48 weeks of PegIFN/RBV at 70% to 85% encourages physicians to recommend the regimens in Taiwan.^12,13^ However, the HCV treatment rate remains low in primary clinic units. High frequency and intensity of AEs with PegIFN/RBV for HCV patients^52^ might cause patients to hesitate to receive therapy and gastrointestinal specialists in primary care units to recommend the therapy.

The reasons for rejecting anti-HCV therapy were fear of AEs in one-third of patients, followed by contraindication because of major hematological or systemic disorders and ineligibility for insurance reimbursement. This finding was in agreement with previous studies.^20,49^ Additionally, we found that older patients resulted in a higher proportion of patients associated with AEs of and ineligibility for IFN-based therapy. IFN/RBV-related AEs comprise a main obstacle for adherence, dose modification, and discontinuation of treatment, especially in the older population.^22^ Future regimens of ultrashort duration (8–12 weeks), IFN-free or IFN/RBV-free would overcome the hesitation at the patient and provider levels.^24,26,29^ Concise pretreatment education and therapeutic counseling during the initial visits is necessary for patients to eliminate fears in the areas in which IFN-free regimens are unavailable.^53^

In the current study, we estimated that 75,410 patients in Taiwan have been treated with IFN-based therapy. The actual number on prescriptions of IFN-based therapy for CHC by reimbursement from Agency of National Health Insurance in Taiwan is 68,438 from 2003 to the end of 2013. The number is close to the estimate in the current study when taking the number of patients with IFN-based therapy by self-pay and those in clinical trials from the year of IFN launched in Taiwan, 1993, to the end of 2013 into consideration.

Recent advances in HCV treatment have led to a significant improvement in treatment efficacy and in eligibility and tolerability. A combination of 2 to 3 DAAs with or without RBV for 8 to 12 weeks could achieve SVR rates of higher than 90% for HCV genotype 1 or 2 patients, whether naive or treatment-experienced and cirrhotic or noncirrhotic.^24,26,29^ However, low disease awareness and treatment rates undermine recent vigorous advances in treatment.^17^ From the estimation in the current study, approximately 0.47 million HCV-viremic patients have not been treated. Increasing the recommendation/acceptance rate to 100% by dramatic IFN-free regimens for those with treatment accessibility could only benefit 50,000 patients, with an improvement of community effectiveness from 10.9% to 19.2% in Taiwan. Strategies for increasing disease awareness and treatment accessibility would be critical issues in the era of newer DAAs. Our findings could provide important information for policy-making at the government and payer levels.

This study demonstrated comprehensive features of HCV treatment in Taiwan. Nevertheless, there were some limitations. First, sampling of the selected gastroenterologists/hepatologists might not represent a complete view of HCV treatment in Taiwan. However, we randomly selected the candidates according to the regional prevalence of HCV infection to minimize bias. Second, special HCV populations, such as uremic patients undergoing maintenance hemodialysis,^33^ major thalassemia patients, and injection drug users,^54^ who have the greatest obstacles to care, were not taken into consideration in the current study. Therefore, the current study might underestimate the gap between the efficacy and effectiveness of HCV treatment. Finally, we did not evaluate the influence of DAA on the model because that the current study was completed before the introduction of DAA to Taiwan (boceprevir in Mar 2014, and telaprevir in Oct 2014). Both DAA should be in combination with PegIFN/RBV with increasing AEs. Currently, no IFN-free DAA regimen was available in Taiwan.

We demonstrated that there is a large gap between clinical efficacy and community effectiveness in HCV treatment, even in a country with favorable host and viral genetics to PegIFN/RBV and well-executed programs in a national health insurance and health care system. In the coming era of IFN-free DAA regimens with short duration, easy dosing, high potency, high genetic barriers, few AEs and drug–drug interaction, there is a clear need to close this large gap by establishing governmental mass screening strategies, educational programs at the patient and provider level, and a more simple-to-access healthcare policy at the payer level.

## Acknowledgments

*We are grateful for the participation of the 89 gastroenterologists/hepatologists for their help with conducting the study*.
